# Society and community: the role of the Netherlands Society of Cardiology

**DOI:** 10.1007/s12471-014-0530-3

**Published:** 2014-02-27

**Authors:** N. M. van Hemel

**Affiliations:** Department of Cardiology UMC Utrecht, Utrecht University, Utrecht, the Netherlands

The earliest available working document of the Netherlands Society of Cardiology, prepared in 1951 [1], declared that the objectives of our Society, which was founded in 1934, were ‘the promotion of the knowledge of cardiovascular diseases and the support of measures in the fight against these diseases, and secondly promotion of the professional interests of the members’. In the notarial articles of 1977 [2], these objectives remained unchanged, except for the one on supporting measures in the fight against cardiovascular diseases, which had disappeared. Thirty years later, our Society articles (2007) show that the objectives now have a wider horizon, namely the development of cardiology and vascular medicine, the promotion of quality of care and the economic and social benefits of the members. It is assumed that these three pillars of Society policy are and will be able to cope with the requirements and questions emerging in the changing conditions of our time and the next decades. Consider for example the implementation of ‘hospital office hours’ versus the former 24 h/7 day duties to reduce fatigue in physicians and residents [3], the growing female emancipation in medicine [4], and the expending influence of patient organisations, health authorities, insurance companies and specifically the media. These factors not only affect the Society as an institute and organisation but also the cardiology community defined as its individual members spread out over the country. Intense mutual understanding and interaction is needed from both the Society and the local cardiology community. At the occasion of the 80th birthday of our Society, I prefer to address two supportive topics for our Society because I would regret if our Society were to become a ‘bloodless’ organisation, comparable with the crumbling Dutch trade unions due to the disinterest of their members.

In the area of development of cardiology and vascular medicine, our Society has reduced its steering role in creating facilities for cardiovascular presentations of Dutch origin at the bi-annual national congresses; these are clustered between professional business and quality of care discussions. It is taken for granted that our Society is not actively involved in complex cellular, experimental or human studies. The independent cardiology departments of universities, fighting for their impact factor, and large general hospitals are better equipped to embark alone or together with the Netherlands Heart Foundation, ICIN-Netherlands Heart Institute, or drug and device companies on this type of (multicentre) study. I doubt whether this policy promotes warm feelings from the members for our Society. For most of those working outside large cardiology centres, Dutch cardiology research is a no-go area. Because the Society manages several large national patient data registries for cardiology specialities [5], the opportunity exists to encourage ‘applied clinical studies’ with the accumulated data which, according to Chalmers et al., provides more impact on care than most experimental research [6]. The cardiology community would undoubtedly profit if members nationwide were to participate in this type of clinical study. The national Followpace study with 23 participating Dutch centres resulting in many insights towards modern long-term conventional cardiac pacing constitutes a nice example of a large and long clinical study based on cooperation and low costs [7]. Daily bedside and outpatient practice in conjunction with involvement in a clinical study strongly promotes the quality of patient care and collegial assessment, such as I experienced for years. Our Society can take the necessary initiatives for these studies.

In the past three decades our Society has devoted major efforts to improving the professionalism of the cardiology community and thereby increasing the quality of care. The implementation of a national cardiology training program in the 1990s, the postgraduate educational CVOI programs and guidelines that almost completely cover cardiac care have equipped our Society to respond adequately to increasing demands from Dutch society in general. These achievements rely on the long-term contribution of many members and deserve applause. However, after several assessments of the quality of departments and thus of the offered care, I could conclude that not the skill and knowledge of physicians but rather the lack of daily communication most often underlies professional shortcomings. Individual positive attitudes to care and compassion with the patient are indispensable but cannot compensate for insufficient communication in the ward and office. The origin of the saying ‘who is my doctor in charge?’ cannot only be attributed to the obliged shortening of the working hours—which has, however, promoted female participation in cardiology—but also to inadequate patient counselling and too few discussions with colleagues in the local cardiology community.

As our Society rightly strives to preserve the role of ‘chef’ of our cardiovascular care menu, communication with all members and other involved parties should be more focused, otherwise the organisation will be put at risk. In view of our increasingly individualised world, communication cannot escape from receiving more attention in the initial and postgraduate cardiology training. This cultural change can result in a better cooperation between our Cardiology Society and its members and our patients will profit from this development.
